# The health beliefs, dengue knowledge and control behaviors among internally displaced persons versus local residents in Kachin Special Region II, Myanmar

**DOI:** 10.1371/journal.pntd.0008321

**Published:** 2020-06-25

**Authors:** Jian-Wei Xu, Hui Liu, Bi Yaw, Hkawn Shawng Nbwi

**Affiliations:** 1 Yunnan Institute of Parasitic Diseases, Yunnan Provincial Centre of Malaria Research, Yunnan Provincial Key Laboratory of Vector-borne Disease Control and Research, Yunnan Provincial Collaborative Innovation Center for Public Health and Disease Prevention and Control, Pu’er City, China; 2 Institute of Pathogens and Vectors, Basic Medical College, Dali University, Xiaguang District, Dali City, China; 3 Laiza City Hospital, Laiza Town, Kachin Special Region II, Myanmar; University of Heidelberg, GERMANY

## Abstract

Dengue fever (DF) is one of main public health problems along the China-Myanmar border, however, data about DF is still lacking in Kachin Special Region II (KSR2), Myanmar. To understand health beliefs in general, and knowledge and treatment-seeking and prevention behaviors related to DF among the neglected population, the study was carried out by using a combination of quantitative household questionnaire surveys (HHSs) and qualitative semi-structured in-depth interviews (SDIs). The HHS questionnaire was administered to a total of 258 household heads. The 215 (83.3%) HHS respondents believed in Christianity and Catholicism. However, the 141 (54.7%,) of the total respondents thought that people with evil practices might be punished by diseases. More respondents believed that too rainy weather and water were more related to disease in the internally displaced person (IDP) camp than the local community (P<0.01). Most of the HHS respondents had sound knowledge of dengue symptoms, causes, vectors, transmission and prevention. The 257 (99.6%) HHS respondents reported that their families went to the public health facilities first to seek treatment. The 210 (84.1%) respondents reported that they turned containers upside down within five days. The key informants (n = 18) identified that the appropriate knowledge and behaviors were attributable to formal school education and specific health education campaign during the outbreak response in 2017, and that Kachin people enjoy conversing with each other, neighbors talked about the dengue information they received. The study results indicated that Kachin people have a good knowledge and behaviors of dengue control. The actual situation of dengue is still not clear due to lacking data of laboratory test. In the context of resources shortage, more international assistance is still needed to promote local dengue control and prevention efforts.

## Author summary

The conflict between the Kachin Independence Army and the Myanmar government armed forces has lasted for more than eight years in the Kachin State (since 9^th^ June 2011). Control of vector-borne diseases is especially important in emergency settings of tropical regions. In 2017, a dengue outbreak occurred and then was successfully controlled in Kachin Special Region (KSR2), Myanmar. To understand further preparedness at the community level for future possible re-emergence of DF, one IDP camp and a local community were sampled to investigate people’s health beliefs in general, knowledge and treatment-seeking and prevention behaviors related to dengue control from August to December, the local dengue transmission season in 2018. This study found that the IDPs and local Kachin residents have sound knowledge about dengue, treatment-seeking and prevention behaviors; an inconsistency exists between their religious and health beliefs due to pressure from various diseases and health problems. The community members perceived DF as a serious and life-threatening disease; however, the local health authority did not think dengue was a priority health problem. The actual situation of dengue is still not clear due to lacking laboratory test and surveillance data. In the emergency context, dengue may still be a local health threat and dengue control is particularly important. The local capacity for dengue laboratory testing and control and prevention activities should be promoted urgently. This should get more international attention and assistance.

## Introduction

The Kachin Independence organization (KIO) signed ceasefires and a peace agreement with the Myanmar government in 1994 following an insurgency that began in 1961. However, the conflict between the Kachin Independence Army (KIA) of the Kachin State and the Myanmar government defense forces broke the agreement on 9^th^ June 2011. As a result of internal conflicts, internally displaced persons (IDPs) from the inland area of Kachin State have lived along the China-Myanmar border for more than eight years [1]. As a consequence of poor sanitation, over-crowding, unclean water, and limited access to health care, the IDPs are particularly vulnerable to infectious diseases, as these environments may foster their transmission [2]. DF is an arbovirus disease transmitted by *Aedes sp*. The World Health Organization (WHO) estimated that there were 284–528 million dengue infections yearly in 128 territories [3, 4]. Presently, DF is one of the main public health problems while malaria has become successfully controlled along the China-Myanmar border [5, 6]. The Laiza City Hospital in Kachin Special Region II (KSR2) of Myanmar detected and reported 127 DF cases by using one step rapid diagnosis test (RDT) of NS1Ag and IgG/IgM in 2017. This is the only available data of dengue incidence in the KSR2. Due to limited coverage of DF surveillance in the KSR2 and the fact that about 75% of dengue infections have no clinical manifestation [7], this figure is probably not indicative of the true dengue situation in the KSR2. DF is still one of the major tropical diseases and an increasing threat to public health in the world [7, 8], but the impact of dengue on local public health is still being neglected now. While extensive investigations about malaria have been done in the KSR2, Myanmar [2, 3, 9–18], studies on DF are still unavailable.

*Aedes* mosquitoes are transmitting vectors of dengue virus, and the main *Aedes* mosquito productive habitats are water containers and discarded tires [19]. The weather, people’s general health beliefs, dengue knowledge and human behavior influence the population dynamics of *Aedes* mosquitoes, and then further influence transmission of dengue virus [20]. To understand health beliefs in general, and knowledge and treatment-seeking and prevention behaviors of this neglected tropical diseases among the neglected population in the KSR2 of Myanmar, this study was carried out during the local dengue transmission season from August to December 2018. To triangulate the study outcomes and obtain in-depth knowledge for comparing any possible differences between the IPDs and local people, mixed methods, including qualitative semi-structured in-depth interviews (SDIs) and quantitative household questionnaire surveys (HHSs) were applied to collect data related to the Kachin people’s health beliefs, dengue knowledge, treatment-seeking and prevention behaviors on dengue in Hpun Lum Yang IDP camp and Laiza Town, KSR2 of Myanmar.

## Methods

### Design and sample size

The design was a cross-sectional study which included quantitative HHSs and qualitative SDIs. The IDP camp and a local community were selected as study sites. Semi-structured in-depth interviews (SDIs) were conducted with nine key informants each in the IPD camp and the local community for a total of 18 SDIs. The intended sample size of the HHSs was calculated using a 5% precision, a 95% confidence interval of the standard value normal distribution and an estimated 20% of household heads who know that mosquitoes transmit dengue virus [21]. The calculated result was a total of 250 households for the HHSs, with individuals sampled equally in the IPD camp and the Laiza town.

### Concept definition

People’s health beliefs usually affect their knowledge, treatment-seeking and preventive behaviors of diseases. Health beliefs are the general perceptions of elements related to health and disease, not just special beliefs about dengue, i.e., people’s perception of the effect of religious, socioeconomic and natural elements on their health and their perception of disease causes. More specifically, respondent’s knowledge regarding DF include their knowledge of the clinical symptoms, perceived risk of contracting DF, treatment-seeking, disease vectors, dengue virus transmission and preventive methods [5].

### Study site and population

All study participants were of the Kachin tribe. The civil war broke out on 9^th^ June 2011. Shortly thereafter, fleeing populations resettled in camps by the border areas. About one month (July–August 2011) after the first conflict, three camps which caught about a population of 12,000 were established along the Myanmar–China border. The Hpun Lum Yang IDP camp with about five thousand IDPs is one of the three camps. The local residents mainly live in Laiza town with a population of about six thousand. The two study sites are located in the same valley with an altitude from 230 to 260m, KSR2, Myanmar, and two sites have similar landscape and ecological environments. Hot temperature, adequate precipitation and lush forest fit for the growth and reproduction of mosquitoes and also for the transmission of vector-borne diseases [9, 12, 13]. There is a clinic that cannot do any laboratory diagnosis test for dengue in the IDP camp. The Laiza City Hospital is the only health facility that can detect dengue with RDTs in the area. Both the clinic in IDP camp and the Laiza City Hospital provide free diagnosis and treatment for the IDP and local residents.

### Household questionnaire survey

The household was the unit of sampling for this survey. The survey instrument was developed in Chinese for a previous study in Shan Special Region IV (SR4), Myanmar [5]. The family wealth index (FWI) was assessed by the household’s physical assets, such as housing, walls, roofs, and transportation means, and was categorized into five groups (Table 1). The local government of KIO assigns a number to each household in KSR2, so a simple computer randomization was used to select households by using existing household numbers in the two study sites for the survey. Researchers firstly visited every selected household and told the household head about the purpose of this study and related questions that would be asked. When oral informed consent was obtained, the questionnaire was administered to each household head. Investigators who understood both the Kachin ethnical language and Chinese from the Laiza City Hospital asked every question in the Kachin ethnical language and then filled out the questionnaire in Chinese [5, 12, 22].

**Table 1:** Principal components for the construction of the family wealth index (FWI).

Family wealth index	Housing characteristics	Transportation tools	Family belongings
1 Most poor	Bamboo walls and sheet iron roofs	None	None or chickens
2 Mid low	Wood walls and sheet iron roofs	Bicycles	Pigs or goats
3 Middle	Brick walls, wood girders and terracotta roofs	Motorcycles	Cattle or horses
4 Mid high	Brick concrete walls and terracotta roofs	Tractors	TV sets or refrigerators
5 Least poor	Steel and concrete	Cars	Shops or elephants

### Semi-structured in-depth interviews

The SDI guideline was developed and 18 key informants were interviewed [5], with nine from each of the two study sites. Interviewees were composed of two clergymen, two health workers, three village leaders and two village representatives who were selected by the other villagers at each of the two study sites. The topics that were discussed included villager beliefs including religion and the causes of diseases, local health problems, especially local dengue situations; knowledge, treatment-seeking and prevention behaviors and their potential reasons [5, 23]. Investigators who understood both the Kachin ethnical language and Chinese from the Laiza City Hospital conducted the interviews in Kachin ethnical language and translated answers to Chinese researchers who took notes.

### Data analysis

Quantitative data were entered in Excel 2007 and were analyzed in Epi Info 7.2 (Centers for Disease Control and Prevention, USA). The percentage and its 95% confidence interval (CI) were computed for each health belief, knowledge item, and treatment seeking and prevention behavior. The percentage of each health belief, knowledge item and behavior between the IDP camp and the local community was compared by chi-squared test. Microsoft Office Excel 2007 was used to enter data from the SDIs. The records were coded according to the contents of the questions and then entered into cells in Microsoft Office Excel 2007. The same content records were combined by code sequencing. The records of each content were independently analyzed by two researchers to generate themes. The two researchers then compared and discussed their findings to finalize the findings [5, 24].

### Ethics

The study was approved by the Department of Health of the KIO, Myanmar, and ethical approval was granted by the Ethics Committee of the Yunnan Institute of Parasitic Diseases in China. Verbal consent was approved by the Ethics Committee as an acceptable form, as the study was interview-based and did not include any human specimens. All study participants were 15-years of age or older. According to the World Medical Association Declaration of Helsinki, the purpose and procedures of the study were explained and disclosed to the participants before obtaining their consent. Participation was entirely voluntary, and participants could pass on a question, take a break or withdraw their consent from the study without providing any explanation at any time. Their continued consent was assumed if they did not refuse to answer questions.

## Results

### Demographics of the HHS and SDI participants

A total of 137 household heads from the IDP camp and 121 from the local community participated in the HHS, and all completed questionnaires were assessed as valid. The mean age of the respondents was 30-years-old (median: 28.0, range: 15–79). The ages of household heads were younger in the IDP camp than in the local community (P = 0.002), mean age 28.2-years-old (median: 26.0, range: 15–79) in the IDP camp versus 32.1-years-old (median: 30.0, range: 15–69) in the Laiza Town. The gender proportions were similar (P = 0.473), overall proportion of male household heads was less than one fourth in the two sites. About half the respondents reported 4–6 years of formal school education, and the proportion of respondents who reported seven or more years of school education was higher in the IDP camp than in the Laiza town (P<0.0001). Most of these households were poor with a family wealth index (FWI) of 1–3. The IDP respondents were poorer than local respondents who have their own houses (Table 2). The 18 key informants of the SDI included seven males (four from the IDP camp and three from the local community) and eleven females (five from the IDP camp and six from the local community), ranging from 26 to 69-years-old. Results of the SDI found that most of the male young adults were recruited into armed forces, so most of the household heads were young females. The on-going conflict has led to change of demographic characteristics and socioeconomic vulnerability. The protracted displacement resulted in significant impacts on livelihoods at the household level. Male adults who were not recruited into armed forces also had to look for work out of the camp. This has resulted in women and children comprising the majority of the IDP population.

**Table 2:** Results of the household questionnaire survey in Kachin Special Region II, Northeastern Myanmar.

Variables	Total No. (%, 95% CI**), n = 258	No. (%, 95% CI*) IDPs **, n = 137	No. ([%, 95% CI*) local residents, n = 121	P-value
Demographics				
Male household head	64(24.9, 19.7–30.7)	31 (22.6, 15.9–30.6)	33 (27.3, 19.6–36.1)	0.4730
Age of the household head (years)				
15–30	171 (66.3, 60.2–70.0)	103 (75.4, 67.1–82.2)	68 (56.2, 46.9–65.2)	0.0020
31–79	87 (33.7, 28.0–39.9)	34 (24.8, 17.8–32.9)	53 (43.8, 34.8–53.1)	0.0020
School education (years)				
0–3	56 (21.7, 16.8–27.2)	16 (11.7, 6.8–18.3)	40 (33.1, 24.8–42.2)	P<0.0001
4–6	128 (49.6, 43.3–55.9)	62 (45.3, 36.7–54.0)	66 (54.6, 45.2–63.6)	0.1724
≥7	74 (28.7, 23.2–34.6)	59 (43.1, 34.6–51.8)	15 (12.4, 7.1–19.6)	P<0.0001
Family wealth index				
1 Most poor	6 (2.3, 0.9–5.0)	4 (2.9, 0.8–7.3)	2 (1.7, 0.2–5.8)	0.7950
2 Mid low	51 (19.8, 15.1–25.2)	21 (15.3, 9.8–22.5)	30 (24.8, 17.4–33.5)	0.0804
3 Middle	167 (64.7, 58.6–70.6)	105 (76.6, 68.7–83.4)	62 (51.2, 42.0–60.4)	P<0.0001
4 Mid high	34 (13.2, 9.3–17.9)	7 (5.1, 2.1–10.2)	27 (22.3, 15.3–30.8)	P<0.0001
5 Least poor	0 (0, 0–1.4)	0 (0, 0–2.7)	0 (0, 0–3.0)	-
Social and religious beliefs				
Poverty is a cause of ill health	229 (88.8, 84.3–92.3)	112 (81.8, 74.3–87.8)	117 (96.7, 91.8–99.1)	0.0003
People with evil practices may be punished by diseases	141 (54.7, 48.4–60.8)	78 (56.9, 48.2–65.4)	63 (52.1, 42.8–61.2)	0.5101
The God will protect good people	147 (57.0, 50.7–63.1)	90 (65.7, 57.1–73.6)	57 (47.1, 38.0–56.4)	0. 0039
Buddhism	43(16.7, 12.3–21.8)	26 (19.0, 12.8–26.6)	17 (14.1, 8.4–21.5)	0.3726
Catholicism	91 (35.3, 29.5–41.4)	18 (13.1, 8.0–20.0)	73 (60.3, 51.0–69.1)	<0.0001
Christianity	124 (48.1, 41.8–54.3)	93 (67.9, 59.4–75.6)	31 (25.6, 18.1–34.4)	<0.0001
Natural and hygiene perceptions				
All natural factors influence health	251 (97.3, 94.5–98.9)	133 (97.1, 92.7–99.2)	118 (97.5, 92.9–99.5)	0.8676
Environments are associated with diseases?				
Too hot	60 (23.3, 18.3–28.9)	31 (22.6, 15.9–30.6)	29 (24.0, 16.7–32.6)	0.9152
Too cold	16 (6.2, 3.6–9.9)	3 (2.2, 0.5–6.3)	13 (10.7, 5.8–17.7)	0.0010
Too rainy	36 (14.0, 10.0–18.8)	32 (23.4, 16.6–31.3)	4 (3.3, 0.9–8.2)	0.0001
Too forested	114 (44.2, 38.0–50.5)	62 (45.3, 36.7–54.0)	52 (43., 34.0–52.3)	0.8084
Rivers, streams and clear water pools near home	90 (34.9, 29.1–41.0)	59 (43.1, 34.6–51.8)	31 (25.6, 18.1–34.4)	0.0051
Polluted water	125 (48.4, 42.2–54.7)	64 (46.7, 38.1–55.4)	61 (50.4, 41.2–59.6)	0.5412
Poor hygiene	256 (99.2, 97.2–99.9)	136 (99.3, 96.0–100.0)	120 (99.2, 95.5–100.0)	0.5333
Environment benefiting health				
Clean and sound hygiene	93 (36.0, 30.2–42.2)	50 (36.5, 28.4–45.1)	43 (35.5, 27.0–44.8)	0.9759
No polluted water	224 (86.8, 82.1–90.7)	123 (89.8, 83.4–94.3)	101 (83.5, 75.6–89.6)	0.1899
Many flowers, grass and trees around house	147 (57.0, 50.7–63.1)	74 (54.0, 45.3–62.6)	73 (60.3, 51.0–69.1)	0.8084
Good hygiene can reduce diseases	239 (92.6, 88.7–95.5)	128 (93.4, 87.9–97.0)	111 (91.7, 85.3–96.0)	0.7784
Heard about dengue	255 (98.8, 96.6–98.8)	137 (100.0, 97.3–100.0)	118 (97.5, 92.9–99.5)	0.2034
Knowledge of DF symptoms	n = 254	n = 135	n = 119	
Fever	240 (94.5, 90.9–97.0)	123 (91.1, 85.0–95.3)	117 (98.3, 94.1–99.8)	0.0253
Headache	150 (59.1, 52.7–65.2)	70 (51.9, 43.1–50.5)	80 (67.2, 58.0–75.6)	0.0183
Orbital pain	93 (36.6, 30.7–42.9)	49 (36.3, 28.2–45.0)	44 (37.0, 28.3–46.3)	0.9856
Pantalgia	136 (53.5, 47.2–59.8)	79 (58.5, 49.7–66.9)	57 (47.9, 38.7–57.2)	0.1170
Rash	14 (5.5, 3.0–9.1)	4 (3.0, 0.8–7.4)	10 (8.4, 4.1–14.9)	0.1051
Others	6 (2.4, 0.9–5.1)	3 (2.2, 0.5–6.4)	3 (2.5, 0.5–7.2)	0.8547
Perceived risks				
Easy to contract dengue	122 (47.3, 41.1–53.6)	53 (38.7, 30.5–47.4)	69 (57.0, 47.7–66.0)	0.0048
Not easy or impossible to get DF	110 (42.6, 36.5–48.9)	70 (51.1, 42.4–59.7)	40 (33.1, 24.8–42.2)	0.0052
A serious illness	254 (98.4, 96.1–99.6)	134 (97.8, 93.7–99.5)	120 (99.2, 95.5–100.0)	0.7042
A deadly disease	253 (98.1, 95.5–99.4)	134 (97.8, 93.7–99.5)	119 (98.3, 94.2–99.8)	0.7103
Do not know or no response	26 (10.1, 6.7–14.4)	14 (10.2, 5.7–16.6)	12 (9.9, 5.2–16.7)	0.8890
Transmissibility	n = 255	n = 137	n = 118	
Yes	224 (87.8, 83.2–91.6)	115 (83.9, 76.7–89.7)	109 (92.4, 86.0–96.4)	0.0625
Transmittable from person to person directly	233 (91.4, 87.2–94.5)	118 (86.1, 79.2–91.4)	115 (97.5, 92.7–99.5)	0.0028
Knowledge of dengue causes				
Bacteria	31 (12.0, 8.3–16.6)	22 (16.1, 10.4–23.3)	9 (7.4, 3.5–13.7)	0.0532
Viruses	66 (25.6, 20.4–31.4)	35 (25.6, 18.5–33.7)	31 (25.6, 18.1–34.3)	0.8968
Flies	25 (9.7, 6.4–14.0)	15 (10.9, 6.3–17.4)	10 (8.3,4.0–14.7)	0.6055
Mosquitoes	206 (79.8, 74.4–84.6)	108 (78.8, 71.0–85.3)	98 (81.0, 72.9–87.6)	0.6661
Animals	7 (2.7, 1.1–5.5)	7 (5.1, 2.1–10.2)	0 (0, 0–3.0)	0.0326
Be rained or shower with cold water	12 (4.7, 2.4–8.0)	12 (4.7, 2.4–8.0)	0 (0, 0–3.0)	0.0024
Eat improper or dirty food	12 (4.7, 2.4–8.0)	9 (6.6, 3.0–12.1)	3 (2.5, 0.5–7.1)	0.2075
Others	36 (14.0, 10.0–18.8)	16 (11.7, 6.8–18.3)	20 (16.5, 10.4–24.4)	0.3462
Do not know or no response	55 (21.3, 16.5–26.8)	31 (22.6, 15.9–30.6)	24 (19.8, 13.1–28.1)	0.6933
Knowledge regarding dengue-transmitting mosquitoes	n = 239	n = 133	n = 106	
Piebald or Aedes	203 (84.9, 79.8–89.2)	109 (82.0, 74.4–88.1)	94 (88.7, 81.1–94.0)	0.2070
Biting time				
Day	150 (58.1, 51.9–64.2)	72 (52.6, 43.9–61.1)	78 (64.5, 55.3–73.0)	0.0705
Night	19 (7.3, 4.5–11.3)	15 (11.0, 6.2–17.4)	4 (3.3, 0.9–8.3)	0.0351
24 hours	45 (17.4, 13.0–22.6)	32 (23.4, 16.6–31.3)	13 (10.7, 5.9–17.7)	0.0124
Do not know or no response	44 (17.1, 12.7–22.2)	18 (13.1, 8.0–20.0)	26 (21.5, 14.5–29.9)	0.1066
Habitats of dengue-transmitting mosquito larva				
All water sites	244 (94.6, 91.1–97.0)	131 (95.6, 90.7–98.4)	113 (93.4, 87.4–97.1)	0.6070
Watered containers or small-scale ponds	246 (95.3, 92.0–97.6)	129 (94.2, 88.8–97.4)	117 (96.7, 91.8–99.1)	0.5040
Do not know or no response	12 (4.7, 2.4–8.0)	8 (5.8, 2.6–11.2)	4 (3.3, 0.9–8.2)	0.5040
Knowledge regarding reducing dengue-transmitting mosquito breeding sites	n = 257	n = 137	n = 120	
Maintain sound hygiene	89 (56.7, 48.6–64.6)	50 (36.5, 28.4–45.1)	39 (32.5, 24.2–41.7)	0.5889
Turn containers upside down	255 (99.2, 97.2–99.9)	135 (98.5, 94.8–99.8)	120 (100.0, 97.0–100.0)	<0.0001
Drain small-scale ponds	27 (17.2, 11.6–24.0)	7 (5.2, 2.1–10.2)	20 (16.7, 10.5–24.6)	0.0049
Others	12(4.7, 2.4–8.0)	4 (2.9, 0.8–7.3)	8 (6.7, 2.9–12.7)	0.2610
Do not know or no response	2 (0.8, 0.1–2.8)	1 (0.7, 0.02–4.0)	1 (0.8, 0.02–4.6)	0.5370
Knowledge regarding preventing dengue-transmitting mosquito bites				
Door and window screens	45 (17.4, 10.4–22.6)	26 (19.0, 12.8–26.6)	19 (15.7, 9.7–23.4)	0.5978
Use of mosquito coils	136 (52.7, 46.4–58.9)	75 (54.7, 46.0–63.3)	61 (50.4, 41.0–59.6)	0.5684
Fogging and spraying with insecticides	139 (53.9, 47.6–60.1)	59 (43.1, 34.6–51.8)	80 (66.2, 57.0–74.5)	0.0003
Use of bed nets	153 (59.3, 53.1–65.4)	66 (48.2, 39.6–56.9)	87 (71.9, 63.0–79.7)	0.0002
Do not know or no response	1 (0.4, 0–2.1)	0 (0, 0–2.7)	1 (0.8, 0.02–4.5)	0.9504
Treatment-seeking behaviors				
Self medication	1 (0.4, 0.01–2.1)	0 (0, 0–2.7)	1 (0.8, 0.02–4.5)	0.9504
Public health facilities	257 (99.6, 97.9–100)	137 (100.0, 97.3–100.0)	120 (99.2, 95.5–100.0)	0.9504
Prevention behaviors				
Turn containers upside down within five days	210 (84.1, 76.1–86.0)	118 (86.1, 79.2–91.4)	92 (76.0, 67.4–83.3)	0.0549
Clean houses every day	111 (43.0, 36.9–49.3)	69 (50.4, 41.7–59.0)	42 (34.7, 26.3–43.9)	0.0160
Clean house surroundings every day	118 (45.7, 39.5–52.0)	69 (50.4, 41.7–59.0)	49 (40.5, 31.7–49.8)	0.1435
Use repellent outdoor	136 (52.7, 46.4–58.9)	75 (54.7, 46.0–63.3)	61 (50.4, 41.2–59.6)	0.5684
Use bed nets	152(58.9, 52.6–65.0)	65 (47.4, 38.9–56.1)	87 (71.9, 63.0–79.7)	0.0001

Note: For all variables, there were a total of 258 respondents (137 IDPs, 121 local residents), unless otherwise indicated.

*95% CI = 95% confidence interval;

** IDP = internally displaced person.

### Themes identified from qualitative study

All participants of the study are Kachin people. The analysis of qualitative data identified five themes for both the IDP and local residents: 1) inconsistency between their religious and health beliefs, 2) people having sound knowledge about dengue because of their received formal school education in childhood and health education in response to the DF outbreaks in 2017 and also their preferring chatting in their spare time in their traditional habit, 3) people only able to seek free treatment from public health sector due to poverty, 4) people complying with instructions of the local government to conduct environment management for dengue vector control, 5) people like fogging and spraying with insecticides in spite of environmental management usually being regarded by health professionals as the most effective intervention for *Aedes sp* control. The analysis of qualitative data also identified a difference in perception of dengue infection between the IPD and local residents, and differences between health workers and other key informants. All nine key informants from the IDP camp felt that the camp conditions promoted disease transmission and the IDP had a higher risk of dengue transmission. All four key informant health workers did not feel that dengue was a serious disease or one of major health problems because of too many other health problems to be solved in the area. The more detailed results of qualitative study are interspersed with the quantitative data in each section below.

### Health beliefs in general

A total of 215 (83.3%) of the HHS respondents believed in Christianity and Catholicism, 147 (68.4%) of the 215 respondents agreed that the God will protect good people, and 141(65.6%) of them agreed that people with evil practices may be punished by diseases. However, the four key informant clergymen were in agreement that the Bible does not say that “Evil will be recompensed with evil”, noting that the Bible says “Everyone is a sinner” and “Jesus Christ had been punished for everyone. We would not be punished as long as we believe in the God and the Jesus.” Comparison between results of the HHSs and the SDIs documented the inconsistency between their beliefs of health and religions.

A total of 251 (97.3%) respondents of the HHSs believed that all of natural factors influenced people’s health. About half of household heads thought that forests, rivers, streams and clear water pools near home were associated with diseases. There were 256 (99.2%) respondents who also believed that people’s diseases were related to poor hygiene. If there was not polluted water near home, people would be more healthy. There were more respondents of the HHSs in the IDP camp than the local residents who believed that rainy weather (P = 0.0001) and water body (P = 0.0051) were related to disease incidence. All nine key informants from the IDP camp felt that the camp conditions promoted diseases. One interviewee noted “The camp is too crowded” while others highlighted the conditions of the camp (“All the grounds are soil” and “Raining makes the camp muddied and dirty”) as contributing to disease transmission (“These are easy to cause diseases.”). Results of the HHSs showed that 147 (57.0%) of the HHS respondents thought that many flowers, grass and trees around houses would benefit health (Table 2). Most of the key informants agreed that their fellow residents in both the IDP camp and the local community did not know that bamboo and tree stump holes, even leaf axils of some plants, could be the breeding habitats of dengue mosquitos, and their fellow residents just thought that flowers, grass and trees could make people happy and healthy.

### Dengue knowledge

A total of 255 (98.8%) of the HHS respondents had heard about dengue. They knew that fever was the basic symptom of DF. There was a higher proportion of the household heads who knew fever and headache symptoms of DF in the IDP camp than in the local community (P<0.05). However, less than 50% of the HHS respondents knew the more specific symptoms of DF such as orbital pain, pantalgia and rash. Most of the HHS respondents perceived DF as a serious illness or a deadly disease (Table 2). All 18 key informants confirmed that there were dengue outbreaks in their communities in 2017, and they received information, education and communication (IEC) about dengue from the health sector during response to the outbreaks. Fewer participants of the HHS in the IDP camp thought that they could easily contract dengue than in the local community (P = 0.0048) (Table 2). However, all nine key informants from the IDP camp thought that the crowded camp could easily promote diseases transmission, so they were at a higher risk of dengue infection. One key informant said “People move frequently out and in the Camp. Young adults are soldiers of the armed forces who live in forests, but they often come back to see their wives, children and parents. Some people go out of the camp working at other parts of Kachin State or even the south of Myanmar and Yunnan Province of China. They may bring the diseases into the camp”. All four key informant health workers from both the IDP camp and local community did not feel that dengue was a serious disease or one of the major health problems. The four health workers agreed that there were more pressing health problems to be solved than dengue in the area.

A total of 224 (87.8%) HHS respondents knew that DF was communicable; and 233 (91.4%) of them felt that it was transmittable from person to person directly by physical contact, speaking and breathing, etc. There were more participants who thought dengue was directly transmittable from person to person in the local community (97.5%) than the IDP camp (86.1%). Only 66 (25.6%) knew that DF was a viral disease, and there was no significant difference about what cause the dengue between the IPDs and the local residents (P = 0.8968). A total 206 (79.8%) of 258 HHS respondents confirmed that dengue was transmitted by mosquitoes, further, 203 (84.9%) of 239 HHS respondents who answered the vector question knew that piebald or *Aedes* were dengue-transmitting mosquitoes; 150 (58.1%) asserted that *Aedes* bit in the daytime; 246 (95.3%) reported that water containers or small-scale pond water were the principal breeding sites for the *Aedes* larvae; 255 (99.2%) regarded that turning containers upside down could help to reduce dengue transmitting mosquitoes to prevent dengue transmission. About half of the HHS participants thought that adult mosquito interventions including fogging and spraying with insecticides, use of mosquito coils and of bed nets could prevent dengue infection (Table 2). These results of the HHS demonstrate that Kachin people had a good knowledge regarding dengue vectors and transmission. Key informants noted several reasons for this sound dengue knowledge, including, 1) most of the Kachin people (>80%) had formal school education and could read in Kachin language; 2) the residents received IEC including posters, leaflets and lectures about dengue during the dengue outbreaks in 2017; 3) the Kachin people have a traditional habit that they enjoy chatting in their spare time, and dengue was one of topics that they talked about.

### Treatment seeking behaviors

Among the HHS respondents, only one (0.4%) household head said that he took drugs himself at first when he had fever. The 257 (99.6%) others said that they would go the public health facilities for treatment first (Table 2). The same result was obtained from the SDI. A key informant said, “Department of Health of the KIO provides medication free for all people regardless of ethnicities and nationalities”. Another noted that “People are poor. They cannot pay for drugs and services of private drug outlets, clinics and hospitals”. In the IDP camp, people sought free treatment in the public clinic first. When they were not getting well, they sought treatment from the Laiza City Hospital. If they did not become well, a few patients might seek treatment from the private sector or hospitals in China if their family had funds, however, most families (>90%) did not have money to do so. Key informants also noted that that public health sector only had limited drugs, equipment and services for primary health care. It was reported that the Laiza City Hospital did not have enough RDTs for dengue detection, so the number of dengue cases diagnosed by laboratory test was limited.

### Prevention behaviors

A total of 210 (84.1%) HHS respondents reported that they turned containers upside down within five days. Around a half of them said that they used mosquito coils, cleaned their houses and surroundings every day. There was no significant difference in these prevention behaviors between the IDPs and local residents. There was a higher proportion of the HHS respondents who reported use of bed nets at the IDP camp than the local community (P = 0.0001) (Table 2). Results of the SDI expanded upon these prevention behaviors. All key informants agreed that behavior change communication (BCC) was carried out in order to respond to the dengue outbreaks in 2017, and people have complied with instructions of the local government about dengue control interventions. One key informant mentioned that “The malaria program delivered a lot of bed nets, but some of the nets are broken and cannot prevent mosquito bite effectively”. It was also noted that residents like fogging and spraying with insecticides. One key informant health worker from the local community said, “Insecticides can kill pests including mosquitoes and flies to prevent diseases. This year (2018), with help from China, we have conducted six rounds of fogging and spraying with insecticides. Now there is not any case of locally infected dengue, only two imported DF patients who came back from Myitkyina.”

## Discussion

The IDPs are particularly vulnerable to infectious diseases, however, health services for them is mostly neglected [3, 25, 26]. Dengue fever is one of the main tropical diseases and increasing threats to public health in the world, however, interventions for DF control has not received enough attention and investment at present [7, 8]. Overcrowded suboptimal living conditions placed the IPDs at an increased risk of infectious diseases [3, 25]. The DF outbreaks in 2017 demonstrated the high risk of dengue transmission in the KSR2. This study investigated health beliefs in general, and knowledge and treatment-seeking and prevention behaviors related to DF among Kachin people including IDPs and local residents, to provide data for planning future dengue interventions in KSR2, Myanmar.

Control of *Aedes sp* with community involvement is regarded as the effective dengue intervention because of the unavailability of anti-dengue virus drugs and low efficacy of current dengue vaccines [19, 27]. More prompt and proper treatment seeking for suspected DF can help reduce transmission and improve patient prognosis. People’s religious and health beliefs can influence their attitudes to dengue vector control and seeking treatment in time [5]. The results of this study showed that most of the HHS respondents believed that people’s diseases were related to poor hygiene. In spite of inconsistency between their religious and health beliefs, more than half of HHS respondents believed that the God will protect good people, and people with evil practices may be punished by diseases. These health beliefs may be attributable to their living conditions and concerning health threats. Overcrowded suboptimal living conditions and limited health service placed the IPDs and local residents feeling at an increased risk of infectious diseases [25]. These health beliefs, on the other hand, may encourage people’s involvement of environment management to clean their surroundings for *Aedes sp* control.

A similar study was completed in the SR4 where there has been peace in the past 30 years. However, Shan people’s knowledge level about dengue was not as good as in the KSR2 because of a lack of proper primary education and IEC campaigns in the SR4 [5]. China has a high political commitment to control of infectious diseases [27]. In order to reduce dengue risk at the common border, China provided insecticides and technical support including health education on dengue to respond to the outbreaks in 2017. This supported successful interventions in dengue control and also promoted dengue knowledge among the IPDs and local residents. The formal school education that most Kachin people received and the traditional habit that Kachin people enjoy conversing with each other, could also help remember and pass on knowledge about dengue each other. All contributed to this good level of knowledge.

People’s perception and awareness of a disease influence their prevention and treatment seeking behaviors [22]. In the KSR2, despite that IDPs and local residents perceived dengue as a serious or deadly disease, only 122 (47.3%) of 258 HHS respondents thought that they could easily contract dengue. However, both the HHS and the SDI participants identified that the IDPs are at a higher risk of dengue infection. In Northern Myanmar, five Special Regions are mostly administered by local ethnic minority authorities along the China-Myanmar border. As a result, health services provided by the Myanmar central government cannot fully cover these regions, and thereby health services there are somewhat limited [5, 12, 22]. In the emergency situation, the Kachin people and especially the IDPs faced many health problems such as a high burden of malaria [1, 9]. DF might not be regarded as one of the top priority health issues due to the shortage of laboratory tests, which might lower people awareness and sensitivity to dengue. This shows that the IDPs are badly in need of international supports.

Treatment seeking behavior is a part of disease control behaviors. As lacking effective antiviral therapies for DF [28], early diagnosis and timely treatment benefit the prognosis of DF patients. In contrast, delay in proper treatment could lead to complications or to severe dengue, and also further transmission [29, 30]. In the context that people were unable to pay for medication by themselves, the free public health facilities were their sole option. In the KSR2, all the HHS respondents sought treatment from the public health service structure at first. However, the public health facilities cannot effectively perform testing for common infectious diseases besides malaria because that lack technicians, equipment and supplies. Since 2007, the Global Fund to Fight AIDS, Tuberculosis and Malaria has continuously supported malaria interventions in the KSR2. The international investment effectively reduced malaria burden and maintained the ability of parasite-based diagnosis of malaria. This also ensured the availability of sound surveillance data about malaria in the KSR2 [6, 9]. Dengue is one of the most neglected tropical diseases in the world [26, 31, 32]. The disease is more neglected among the IDPs [31]. Despite that most suspected DF patients seek treatment from public health facilities, limited laboratory tests for dengue led to unavailability of sufficient data that could show an epidemiological profile of dengue in the KSR2. Control of communicable diseases is particularly important in the IDP camps as these environments may foster the re-emergence of previously controlled diseases. In spite of successful dengue prevention in 2018, adequate health services are still in urgent need for surveillance, prevention and treatment of dengue in the KSR2.

In the IDP camps and settlements, incidence of infectious disease can be effectively reduced if preventive measures are conducted timely and properly [33]. Results from the HHSs and the SDIs demonstrated that effective interventions were implemented to respond to the dengue outbreaks of 2017 and to prevent dengue in 2018. Most of the families turned containers upside down in five days, and about half of them cleaned their house and surroundings, used mosquito coils and bed nets every day. Small water ponds, water containers and discarded tires are the breeding sites for *Aedes* mosquitos. In a long term view, environmental management is the most important strategy for vector control to prevent dengue [5, 19, 34]. The SDI identified that both the IDPs and local residents like spraying with insecticides. The appropriateness of six rounds of fogging and spraying with insecticides in 2018 deserves to be discussed further. Is it necessary or overuse of insecticides? In response to emergencies and foci of vector borne diseases, fogging and spraying with insecticides can usually be used, however, it might not be appropriate for routine prevention. In the setting of the IDP camp, fogging and spraying with insecticides might be needed to solve other health problems, but it is necessary to further investigate.

This study was unavoidably limited by two obvious weaknesses. First, this study was planned to be quantitatively dominated, with use of qualitative data to triangulate the quantitative results and to explore reasons for the quantitative results. While the investigators are not experts in qualitative research methods, the semi-structured in-depth interview guide questions were open-ended and the authors think the data obtained achieved the study purpose. Second, the same standards for the family wealth index (FWI) are applied to both the IDP and the local community. The same standards of FWI could lead to false results of the previous wealth of the IPDs before fleeing from their homes. However, the IPDs lost their houses and other properties that cannot be moved in conflicts. The FWI of the IDP would be an accurate reflection of their present situation.

In conclusion, dengue is perceived as a higher risk in the IPD camp than the local community. Both the IPDs and local residents have sound knowledge, treatment seeking and prevention behaviors. Their present health beliefs and knowledge benefit dengue control behaviors. In particular, people reported seeking treatment for dengue first from the public health service structure in the KSR2. The actual situation of dengue is still not clear due to lacking data of laboratory tests, but dengue may still be one of the local health threats. In the context of emergency and resources shortage, more international assistance is still needed to promote the local ability to conduct the dengue laboratory test, control and prevention.

## References

[1] Zhou G, Lo E, Zhong D, Wang X, Wang Y, et al: Impact of interventions on malaria in internally displaced persons along the China–Myanmar border: 2011–2014. Malar J. 2016; 15:471 10.1186/s12936-016-1512-2 27628040 PMC5024476

[2] Anderson J, Doocy S, Haskew C, Spiegel P, Moss W: The burden of malaria in post-emergency refugee sites: A retrospective study. Conflict and Health. 2011; 5:17 10.1186/1752-1505-5-17 21929774 PMC3186739

[3] WHO. Dengue control. Geneva: World Health Organization. http://www.who.int/denguecontrol/epidemiology/en/ Accessed on 1^st^, March. 2019.

[4] Oliveira LNdS, Itria A, Lima EC. Cost of illness and program of dengue: A systematic review. PLoS one. 2019: 14(2): e0211401 10.1371/journal.pone.0211401 30785894 PMC6382265

[5] Xu JW, Liu H, Ai Z, Yu Y, Yu B. The Shan people’s health beliefs, knowledge and perceptions of dengue in Eastern Shan Special Region IV, Myanmar. PLoS Negl Trop Dis. 2019, 13(6):e0007498 10.1371/journal.pntd.0007498 31247022 PMC6619833

[6] Xu JW, Li Y, Yang HL, Zhang J, Zhang ZX, Yang YM, Zhou HN, Havumaki J, Li HX, Liu H, Zhou H, Xie XY, Dong JX, Zhang Y, Sun XY, Li B, Li JY, Tian YH, Wang PY, Li BF: Malaria control along China-Myanmar Border during 2007–2013: an integrated impact evaluation. Infectious Diseases of Poverty. 2016; 5:75 10.1186/s40249-016-0171-4 27507163 PMC4979141

[7] WHO. Global Strategy for Dengue Prevention and Control 2012–2020. Geneva: World Health Organization 2012 (accessed on 17 May 2017).

[8] WHO/TDR. Global Report for Research on Infectious Diseases of Poverty. World Health Organization on behalf of Special Programme for Research and Training in Tropical Diseases. 2010 (accessed on 17 May 2017).

[9] Liu H, Xu JW, Bi Y: Malaria burden and treatment targets in Kachin Special Region II, Myanmar from 2008 to 2016: A retrospective analysis. PLoS one. 2018; 13(4): e0195032 10.1371/journal.pone.0195032 29614088 PMC5882093

[10] Huang F, Takala-Harrison S, Liu H, Xu JW, Yang HL, Adams M, Shrestha B, Mbambo G, Rybock D, Zhou SS, Xia ZG, Zhou XN, Plowe C, Nyunt MM: Prevalence of Clinical and Subclinical *Plasmodium falciparum* and *Plasmodium vivax* Malaria in Two Remote Rural Communities on the Myanmar–China Border. Am. J. Trop. Med. Hyg. 2017; 97(5): 1524–1531. 10.4269/ajtmh.17-0167 29016341 PMC5817756

[11] Xu JW, Liu H: The relationship of malaria between Chinese side and Myanmar’s five special regions along China–Myanmar border: a linear regression analysis. *Malar J*. 2016; 15:368 10.1186/s12936-016-1413-4 27430217 PMC4949750

[12] Liu H, Xu JW, Guo XR, Lin YX, Yu GC, Zhou DL: Coverage, use and maintenance of bed nets and related influence factors in Kachin Special Region II, northeastern Myanmar. Malar J. 2015;14: 212 10.1186/s12936-015-0727-y 25990715 PMC4457094

[13] Liu H, Yang HL, Tang LH, Li XL, Huang F, Wang JZ, Li CF, Wang HY, Nie RH, Li CF, Guo XR, Lin YX, li M, Xu JW: In vivo monitoring of dihydroartemisinin piperaquine sensitivity in Plasmodium falciparum along the China- Myanmar border of Yunnan Province, China from 2007 to 2013. *Malar J*. 2015;14:47 10.1186/s12936-015-0584-8 25652213 PMC4333884

[14] Wang X, Zhou G, Zhong D, Wang X, Wang Y, Yang Z, et al: Life-table studies revealed significant effects of deforestation on the development and survivorship of *Anopheles minimus* larvae. Parasit Vectors. 2016; 9:323 10.1186/s13071-016-1611-5 27267223 PMC4895827

[15] Zhong D, Wang X, Xu T, Zhou G, Wang Y, Lee MC, et al: Effects of microclimate condition changes due to land use and land cover changes on the survivorship of malaria vectors in China–Myanmar border region. PLoS One. 2016;11:e0155301 10.1371/journal.pone.0155301 27171475 PMC4865052

[16] Lo E, Nguyen J, Oo W, Hemming-Schroeder E, Zhou G, Yang Z, et al: Examining *Plasmodium falciparum* and *P*. *vivax* clearance subsequent to antimalarial drug treatment in the Myanmar–China border area based on quantitative real-time polymerase chain reaction. BMC Infect Dis. 2016;16:154 10.1186/s12879-016-1482-6 27084511 PMC4833920

[17] Hu Y, Zhou G, Ruan Y, Lee MC, Xu X, Deng S, et al: Seasonal dynamics and microgeographical spatial heterogeneity of malaria along the China–Myanmar border. Acta Trop. 2016; 157: 1 2–19. 26812008 10.1016/j.actatropica.2016.01.022 PMC4779690

[18] Lo EYY, Zhou G, Oo W, Lee M-C, Baum E, Felgner PL, et al: Molecular inference of sources and spreading patterns of *Plasmodium falciparum* malaria parasites in internally displaced person settlements in Myanmar–China border area. Infect Genet Evol. 2015; 33:189–196. 10.1016/j.meegid.2015.05.002 25952567 PMC4679153

[19] WHO. Dengue Guidelines for diagnosis, treatment, prevention and control. Geneva: World Health Organization 2009 (accessed on 27 May 2016). 23762963

[20] Barrera R, Amador M, MacKay AJ. Population Dynamics of Aedes aegypti and Dengue as Influenced by Weather and Human Behavior in San Juan, Puerto Rico. PLoS Negl Trop Dis. 2011; 5(12): e1378 10.1371/journal.pntd.0001378 22206021 PMC3243685

[21] Israel GD. determining sample size. program evaluation and organization development: institute of food and agricultural sciences, university of florida 1992 https://www.tarleton.edu/academicassessment/documents/samplesize.pdf. (Accessed on 12 Dec 2015).

[22] Xu JW, Xu QZ, Liu H, Zeng YR. Malaria treatment-seeking behaviour and related factors of Wa ethnic minority in Myanmar: a cross-sectional study. Malar J. 2012; 11:417 10.1186/1475-2875-11-417 23237576 PMC3529692

[23] Xu JW, Liao YM, Liu H, Nie RH, Havumaki J. Use of Bed Nets and Factors That Influence Bed Net Use among Jinuo Ethnic Minority in Southern China. PLoS one. 2014; 9(7): e103780 10.1371/journal.pone.0103780 25080267 PMC4117543

[24] Xu JW, Liu H, Zhang Y, Guo XR, Wang JZ. Risk factors for border malaria in a malaria elimination setting: a retrospective case-control study in Yunnan, China. Am J Trop Med Hyg. 2015; 92(3):546–551. 10.4269/ajtmh.14-0321 25601994 PMC4350546

[25] Spiegel PB, Hering H, Paik E, Schilperoord M. Conflict-affected displaced persons need to benefit more from HIV and malaria national strategic plans and global fund grants. Confl Health. 2010; 4:2 10.1186/1752-1505-4-2 20205901 PMC2827465

[26] Kirkby K, Galappaththy GN, Kurinczuk JJ, Rajapakse S, Fernando SD. Knowledge, attitudes and practices relevant to malaria elimination amongst resettled populations in a post-conflict district of northern Sri Lanka. Trans R Soc Trop Med Hyg. 2013; 107:110–118. 10.1093/trstmh/trs015 23222949

[27] Lai S, Huang Z, Zhou H, Anders K, Perkins TA, Yin W, et al The changing epidemiology of dengue in China, 1990–2014: a descriptive analysis of 25 years a of nationwide surveillance data. BMC Medicine. 2015; 13(100): 1–12. 25925417 10.1186/s12916-015-0336-1 PMC4431043

[28] Lindsay S, Wilson A, Golding N, et al Improving the built environment in urban areas to control Aedes aegypti-borne diseases. Bull World Health Organ. 2017; 95:607–608. 10.2471/BLT.16.189688 28804174 PMC5537749

[29] Elsinga J, Lizarazo EF, Vincenti MF, et al Health Seeking Behaviour and Treatment Intentions of Dengue and Fever: A Household Survey of Children and Adults in Venezuela. PLoS Negl Trop Dis. 2015; 9: e0004237 10.1371/journal.pntd.0004237 26624283 PMC4666462

[30] Liu H, Xu JW, Ai Z, et al, Treatment seeking behavior and associated factors of suspected dengue fever among Shan people in eastern Shan special region IV, Myanmar: a cross-sectional study. BMC Health Services Research. 2020; 20:318 10.1186/s12913-020-05163-z 32299436 PMC7164341

[31] Aagaard-Hansen J, Chaignat CL. Neglected tropical diseases: equity and social determinants In: Blas E, Sivasankara Kurup A, editors. Equity, social determinants and public health programmes. Geneva: World Health Organization, 2014:136–157(accessed on 27 May 2018).

[32] Sommerfeld J, Ramsay A, Pagnoni F, Terry RF, Guth JA, et al Applied Research for Better Disease Prevention and Control. PLoS Negl Trop Dis. 2015; 9: e3378 10.1371/journal.pntd.0003378 25568958 PMC4287520

[33] Richards AK, Banek K, Mullany LC, Lee CI, Smith L, Oo EK, et al Cross-border malaria control for internally displaced persons: observational results from a pilot programme in eastern Burma/Myanmar. Trop Med Int Health. 2009;14:512–21 10.1111/j.1365-3156.2009.02258.x 19254232

[34] Fritzell C, Raude J, Adde A, Dusfour I, Quenel P, Flamand C. Knowledge, Attitude and Practices of Vector-Borne Disease Prevention during the Emergence of a New Arbovirus: Implications for the Control of Chikungunya Virus in French Guiana. PLoS Negl Trop Dis. 2016; 10(11): e0005081 10.1371/journal.pntd.0005081 27802275 PMC5089683

